# Perceived risk of HIV transmission by blood transfusion among gay, bisexual, and other men who have sex with men (gbMSM) in Australia

**DOI:** 10.1111/trf.17456

**Published:** 2023-06-10

**Authors:** Luke Gahan, Clive R. Seed, Mohamed A. Hammoud, Garrett Prestage, Veronica C. Hoad, John M. Kaldor

**Affiliations:** ^1^ School of Humanities and Social Sciences, La Trobe University Melbourne Victoria Australia; ^2^ Australian Red Cross Lifeblood Melbourne Victoria Australia; ^3^ Kirby Institute, Faculty of Medicine, UNSW Sydney Sydney New South Wales Australia

**Keywords:** blood donation, deferral policy, gay and bisexual men, gbMSM, HIV, men who have sex with men

## Abstract

**Background:**

In Australia, men who have sex with men (MSM) are deferred from blood donation for 3 months from last sexual contact. Internationally, deferral policies for MSM are evolving in the direction of expanded inclusivity in response to community expectations. To inform future policy options, we assessed perceptions of the risk of HIV transmission from blood transfusion among Australian MSM.

**Study Design and Methods:**

Flux is an online prospective cohort of Australian gay and bisexual men (cis or trans, regardless of their sexual history) and other men who have had sex with men (gbMSM). We included questions on blood donation rules, window period (WP) duration, infectivity of blood from people with HIV on treatment and attitudes to more detailed questioning of sexual practices in the regular survey of Flux participants and conducted a descriptive analysis of responses.

**Results:**

Of 716 Flux participants in 2019, 703 responded to the blood donation questions. The mean age was 43.7 years (SD 13.6 years). Overall, 74% were willing to confidentially respond to specific sexual behavior questions, such as the last time they had sex and the type of sex they had, in order to be considered eligible to donate blood. The majority (92%) of participants correctly assessed the duration of the WP as less than 1 month. When asked whether transfusion of blood from a donor with HIV and an undetectable viral load could transmit HIV, just under half (48%) correctly said yes.

**Conclusion:**

Our study suggests Australian gbMSM are generally comfortable with answering more detailed questions regarding sexual activity during the assessment to donate, indicating they would do so honestly. gbMSM are knowledgeable about the WP duration, important for their ability to correctly self‐assess their HIV risk. However, half of participants incorrectly assessed the transmissibility by blood transfusion from an HIV positive person with an undetectable viral load, suggesting the need for a targeted education campaign.

**Abbreviations**
AIDS: Acquired immune deficiency syndrome
ARV: Anti‐retroviral medications
FLUX: Following lives undergoing change study
gbMSM: Gay, bisexual, and other men who have sex with men
HIV: Human immunodeficiency virus
Lifeblood: Australian Red Cross Lifeblood
MSM: Men who have sex with men
NHMRC: Australian National Health and Medical Research
NBS: US National HIV Behavioral Surveillance survey
NAT: Nucleic Acid Test
PrEP: Pre‐exposure prophylaxis
STI: Sexually transmitted infections
U=U: Undetectable equals untransmittable
UNSW: University of New South Wales
UK: United Kindom
USA: United States of America
WP: Window period

## 1 INTRODUCTION

The policy of indefinitely excluding men who have sex with men (MSM) from donating blood commenced after cases of acquired immune deficiency syndrome (AIDS) were diagnosed in blood transfusion recipients in the United States in 1982.^1^ Prior to widespread adoption of HIV antibody testing of blood donors in 1985, blood authorities internationally sought to minimize the risk of HIV transfusion‐related transmission using screening questions designed to identify individuals at increased risk of HIV.^2^ Male‐to‐male sex was identified as a major route of transmission, leading to indefinite deferral, effectively exclusion from donation in many countries, of all men who reported any history of sex with another man.^2^, ^3^

Exclusion of MSM was routine international practice until 2000, when Australia became the first country to implement a nationally applicable deferral period of 12‐months since last male‐to‐male oral or anal sex. A subsequent risk assessment found no evidence of an increased risk of transfusion‐transmitted HIV in Australia following this change.^4^ Assessments of 12‐month MSM deferrals implemented in UK, Canada, and USA also found that HIV‐risk was essentially unaffected by the reduced deferral period.^5^, ^6^, ^7^

With continuing concern about the deferral policy being unnecessarily restrictive, and the updating of evidence regarding the accuracy of tests, Clackett et al investigated perceptions in an online cohort of gbMSM in Australia. They found while there was strong motivation to donate among participants, the existing 12‐month deferral policy prevented many gbMSM in Australia from doing so. For most, this policy was viewed as discriminatory or unnecessarily strict.^8^ A recent study using analogous methodology among New Zealand gay and bisexual men found very similar attitudes toward their existing 12‐month MSM deferral.^9^

Australian Red Cross Lifeblood (Lifeblood) has responded to concerns about the deferral period for MSM by convening independent advisory panels in 2012 and 2017 to provide strategic recommendations.^10^, ^11^ Submissions by Lifeblood to the Australian regulator (Therapeutic Goods Administration) following recommendations of the 2017 panel led to deferral for MSM being reduced to 3 months in January 2021.^12^

Internationally, deferral policies for higher‐risk sexual activity—including MSM—continue to evolve.^13^, ^14^ Many countries have reduced their “time‐limited” deferrals for MSM, generally from 12 months to either 4 or 3 months (currently the shortest deferral period).^6^, ^15^, ^16^, ^17^, ^18^ Other countries have transitioned to an approach termed “gender‐neutral” or “individualized” risk assessment, which defers donors for recent sexual contact of types determined to be higher‐risk, without specifying the gender of the sexual partners.^13^, ^19^, ^20^, ^21^, ^22^, ^23^ Some countries permit plasma donation by MSM either for clinical transfusion or plasma for fractionation.^24^, ^25^, ^26^

HIV tests used in blood screening are generally extremely sensitive in detecting established infection. The purpose of the sexual activity‐based screening questions is to minimize the risk of donation by individuals with recently acquired infection who are still in the so‐called “window period” (WP) so will test negative because the relevant biological markers are not yet detectable.^27^ The estimated WP has steadily narrowed over time with improving test technology. However, there is recent evidence that rare cases of HIV infection that arise in people using pre‐exposure prophylaxis (PrEP) as HIV prevention have an extended WP.^28^, ^29^ Lifeblood introduced a 12‐month deferral period for whole blood from donors using PrEP^30^ (then reduced to 3 months for plasma^31^), with other countries adopting similar changes.^15^, ^32^, ^33^ Several studies have identified undisclosed HIV anti‐viral treatment and PrEP use among a minority of HIV (positive and negative) blood donors constituting a potential transfusion transmission risk.^32^, ^33^, ^34^, ^35^

To build on the findings of our previous study,^8^ we devised further blood donation relevant questions for inclusion in the same online survey of gbMSM to explore their understanding of HIV‐risk in the context of blood donation. The questions targeted their knowledge regarding: the length of the WP for blood donation tests (5.4–8.5 days^36^ for Lifeblood HIV testing), their willingness to be asked—and honestly answer—specific sexual behavior questions in a confidential interview, and their perception of the infectiousness of blood donated by individuals with an undetected HIV viral load. The latter pertains to the “Undetectable equals Untransmittable” (U=U) global HIV campaign being incorrectly extrapolated to the infectivity of transfused blood products.^37^ We also investigated their attitudes toward potential novel screening questions to inform future HIV‐risk deferral policy development.

## 2 MATERIALS AND METHODS

### 2.1 Study design

The “Following Lives Undergoing Change (Flux) Study” is a national, online, open‐prospective observational cohort of gbMSM in Australia, with the primary objective of examining associations with health‐seeking behaviors. The study protocol has been published previously.^38^ In brief, participants were recruited between September 25, 2014 and July 7, 2018 through gay community websites and online media, Facebook, mobile phone applications, and gay sexual networking websites. We conducted a descriptive, cross‐sectional study of blood donation attitudes and beliefs using the Flux cohort.

### 2.2 Study participants

Men were eligible to participate in the study if they identified as male (cis or transgender); were at least 16 years and 6 months of age; identified as gay, bisexual (regardless of sexual history), or were a man who had sex with a man in the past 12 months; and lived in Australia. At enrolment, participant details and online informed consent were obtained from all participants. Participants verified their enrolment through an email receipt at enrolment. No compensation was offered for participation. Ethical approval was provided by the Human Research Ethics Committee of UNSW Sydney.

### 2.3 Measures

An online questionnaire included demographic items such as age, education, sexual identity, self‐reported HIV serostatus, HIV/STI testing history, and use of anti‐retroviral medications (ARV) including PrEP. At each subsequent survey round, participants were asked to self‐report their HIV serostatus, HIV/STI testing history, use of ARV, and sexual behaviors in the previous 6 months with casual and regular partners.^39^ A hierarchy of HIV risk sexual behavior was based on a previously used classification system, ranging from lowest risk to highest risk, no sexual partners, no anal sex, consistent condom use, insertive‐only condomless anal intercourse, and any receptive condomless anal intercourse.^40^ As a component of an Australian National Health and Medical Research (NHMRC) Partnership Project, additional questions were added on blood donation to the 2019 survey. These included participants blood donation histories and attitudes toward deferral practices for MSM. Participants were also asked to report their knowledge of, and attitudes toward, several statements (see Table 1) related to blood donation and HIV transfusion‐transmission among MSM. Three of these statements were based on corresponding questions added to the 2014 US National HIV Behavioral Surveillance (NBS) survey.^41^

**TABLE 1:** Responses to blood donation questions.

	HIV positive		HIV negative		Total	*p*‐Value
	Not on ARV	On ARV	Not using PrEP	Using PrEP		
*n* (%)	2 (0.3)	49 (7.0)	345 (49.1)	307 (43.7)	703 (100.0)	
Have you ever donated blood?						
No	1 (50.0)	33 (67.3)	235 (68.1)	192 (62.5)	461 (65.6)	.471
Yes	1 (50.0)	16 (32.7)	110 (31.9)	115 (37.5)	242 (34.4)	
Did you have sex with another man in the 12 months before donating blood?						
No	1 (50.0)	10 (20.4)	84 (24.3)	81 (26.4)	176 (25.0)	.778
Yes	0 (0.0)	6 (12.2)	24 (7.0)	31 (10.1)	61 (8.7)	
Did not donate blood	1 (50.0)	33 (67.3)	235 (68.1)	192 (62.5)	461 (65.6)	
Did not answer	0 (0.0)	0 (0.0)	2 (0.6)	3 (1.0)	5 (0.7)	
In a confidential interview to determine your eligibility for blood donation, would you be willing to be asked specific sexual behavior questions, such as the last time you had sex and the precise type of sex you had?						
No	1 (50.0)	18 (36.7)	78 (22.6)	81 (26.4)	178 (25.3)	.461
Yes	1 (50.0)	31 (63.3)	266 (77.1)	225 (73.3)	523 (74.4)	
Did not answer	0 (0.0)	0 (0.0)	1 (0.3)	1 (0.3)	2 (0.3)	
The testing “window period” for detecting new HIV infection is the time period between when the first point of infection and when the infection becomes detectable by available testing methods. Had you heard of the HIV testing window period before now?						
No	0 (0.0)	3 (6.1)	74 (21.4)	34 (11.1)	111 (15.8)	**.005**
Yes	2 (100.0)	46 (93.9)	270 (78.3)	273 (88.9)	591 (84.1)	
Did not answer	0 (0.0)	0 (0.0)	1 (0.3)	0 (0.0)	1 (0.1)	
For the methods currently used to screen donated blood, how long do you think the testing window period for HIV infection is?						
Less than 1 week	1 (50.0)	18 (36.7)	106 (30.7)	91 (29.6)	216 (30.7)	.654
Between 1 week and 1 month	1 (50.0)	27 (55.1)	205 (59.4)	196 (63.8)	429 (61.0)	
Between 1 month and 3 months	0 (0.0)	4 (8.2)	34 (9.9)	20 (6.5)	58 (8.3)	
“U=U” means that a person living with HIV has an undetectable viral load and cannot transmit HIV through sexual contact. If a person living with HIV had an undetectable viral load were to donate blood, do you think their blood could transmit HIV by transfusion?						
No	1 (50.0)	26 (53.1)	148 (42.9)	181 (59.0)	356 (50.6)	**<.001**
Yes	0 (0.0)	22 (44.9)	194 (56.2)	124 (40.4)	340 (48.4)	
Did not answer	1 (50.0)	1 (2.0)	3 (0.9)	2 (0.7)	7 (1.0)	

### 2.4 Analysis

A descriptive analysis of the 2019 Flux cohort donation question responses was conducted. Statistical analysis was conducted using IBM SPSS Statistics 25 (IBM Corp). Demographic items were summarized by HIV status and by use of ARV (HIV positive not on ARV; HIV positive on ARV; HIV negative not on PrEP; HIV negative on PrEP) and expressed as numbers and percentages. Categorical variables were analyzed using Pearson's chi‐square test, and continuous variables were analyzed using *t*‐test. We used Type I error of 5% for these analyses. Participants with missing data included in this analysis were noted accordingly and included in the denominator.

## 3 RESULTS

Of the 716 Flux participants in 2019, 703 responded to the blood donation questions. The mean age was 43.7 years (SD 13.6 years). A majority of the participants were Australian‐born (78.4%), and most (89.6%) identified as gay (Table 2). A majority, 86.9%, reported being HIV negative (*n* = 611), while 7.3% reported being HIV positive (*n* = 51) and 5.8% reported their HIV status as unknown or said they had never been tested (*n* = 41). Of those who reported being HIV negative or unknown HIV status/never tested, 47.1% had used PrEP in the past 6 months (*n* = 307) while 52.9% had not (*n* = 345).

**TABLE 2:** Demographics and sexual behaviors with nonmonogamous partners of surveyed participants (*n* = 703).

	HIV positive		HIV negative		Total
	Not on ARV	On ARV	Not using PrEP	Using PrEP	
*n* (%)	2 (0.3)	49 (7.0)	345 (49.1)	307 (43.7)	703 (100.0)
Age					
Mean (SD)	54.0 (8.49)	53.2 (10.60)	42.2 (14.32)	43.9 (12.52)	43.7 (13.58)
19–25	0 (0.0)	0 (0.0)	39 (11.3)	9 (2.9)	48 (6.8)
26–35	0 (0.0)	4 (8.2)	99 (28.7)	86 (28.0)	189 (26.9)
36–45	0 (0.0)	7 (14.3)	71 (20.6)	85 (27.7)	163 (23.2)
46–55	1 (50.0)	13 (26.5)	63 (18.3)	60 (19.5)	137 (19.5)
56+	1 (50.0)	25 (51.0)	73 (21.2)	67 (21.8)	166 (23.6)
State of residence					
New South Wales	2 (100.0)	21 (42.9)	129 (37.4)	165 (53.7)	317 (45.1)
Victoria	0 (0.0)	12 (24.5)	94 (27.2)	71 (23.1)	177 (25.2)
Queensland	0 (0.0)	10 (20.4)	59 (17.1)	33 (10.7)	102 (14.5)
Northern Territory	0 (0.0)	0 (0.0)	7 (2.0)	2 (0.7)	9 (1.3)
Western Australia	0 (0.0)	2 (4.1)	23 (6.7)	11 (3.6)	36 (5.1)
South Australia	0 (0.0)	3 (6.1)	10 (2.9)	8 (2.6)	21 (3.0)
Australian Capital Territory	0 (0.0)	0 (0.0)	17 (4.9)	10 (3.3)	27 (3.8)
Tasmania	0 (0.0)	0 (0.0)	3 (0.9)	4 (1.3)	7 (1.0)
Did not answer	0 (0.0)	1 (2.0)	3 (0.9)	3 (1.0)	7 (1.0)
Country of birth					
Australia	2 (100.0)	39 (79.6)	280 (81.2)	230 (74.9)	551 (78.4)
New Zealand, Europe & North America	0 (0.0)	5 (10.2)	38 (11.0)	39 (12.7)	82 (11.7)
Asia	0 (0.0)	2 (4.1)	9 (2.6)	25 (8.1)	36 (5.1)
Other	0 (0.0)	3 (6.1)	18 (5.2)	13 (4.2)	34 (4.8)
Highest level of education					
Did not complete high school	0 (0.0)	4 (8.2)	21 (6.1)	8 (2.6)	33 (4.7)
Completed high school	0 (0.0)	3 (6.1)	47 (13.6)	26 (8.5)	76 (10.8)
Trade certificate	0 (0.0)	8 (16.3)	29 (8.4)	42 (13.7)	79 (11.2)
Undergraduate degree	0 (0.0)	16 (32.7)	133 (38.6)	112 (36.5)	261 (37.1)
Postgraduate degree	2 (100.0)	18 (36.7)	115 (33.3)	119 (38.8)	254 (36.1)
Employment					
Full time	1 (50.0)	22 (44.9)	213 (61.7)	218 (71.0)	454 (64.6)
Part time	1 (50.0)	11 (22.4)	42 (12.2)	30 (9.8)	84 (11.9)
Not in workforce	0 (0.0)	16 (32.7)	90 (26.1)	59 (19.2)	165 (23.5)
Sexual identity					
Gay	2 (100.0)	44 (89.8)	308 (89.3)	288 (93.8)	642 (91.3)
Bisexual	0 (0.0)	2 (4.1)	22 (6.4)	13 (4.2)	37 (5.3)
Other	0 (0.0)	3 (6.1)	15 (4.3)	6 (2.0)	24 (3.4)
HIV testing					
Never/not tested in the previous 6 months	0 (0.0)	0 (0.0)	218 (63.2)	9 (2.9)	227 (32.3)
Tested for HIV in the previous 6 months	0 (0.0)	1 (2.0)	127 (36.8)	298 (97.1)	426 (60.6)
HIV positive	2 (100.0)	48 (98.0)	0 (0.0)	0 (0.0)	50 (7.1)
Gay social engagement with other gay men					
Mean (SD)	5.5 (0.71)	4.0 (1.29)	3.3 (1.54)	4.4 (1.45)	3.8 (1.57)
Sex with non‐relationship partners					
No sex with non‐relationship partners	0 (0.0)	18 (36.7)	190 (55.1)	22 (7.2)	230 (32.7)
No anal intercourse with non‐relationship partners	0 (0.0)	4 (8.2)	20 (5.8)	8 (2.6)	32 (4.6)
Anal intercourse (with/without a condom) with non‐relationship partners	2 (100.0)	27 (55.1)	135 (39.1)	277 (90.2)	441 (62.7)
How open are you about having (or not having) sex with men?					
Extremely open	0 (0.0)	24 (49.0)	140 (40.6)	175 (57.0)	339 (48.2)
Somewhat open	1 (50.0)	20 (40.8)	138 (40.0)	104 (33.9)	263 (37.4)
Not very open	1 (50.0)	4 (8.2)	45 (13.0)	24 (7.8)	74 (10.5)
I keep it completely to myself	0 (0.0)	1 (2.0)	22 (6.4)	4 (1.3)	27 (3.8)

A majority, 67.3%, reported having sexual activity in the previous 6 months with a non‐relationship partner (*n* = 473). Of these, 93.2% (*n* = 441) reported having had anal intercourse (with or without a condom), while 6.8% reported having had oral sex only (*n* = 32). When we examine this by HIV status and ARV use, just over half (55.1%) of those who stated they were HIV negative and not on PrEP reported no sex outside of a monogamous relationship in the previous 6 months (*n* = 190). Of the men who reported being HIV negative and on PrEP, 7.2% reported having no nonmonogamous sexual partners (i.e., no sex or only monogamous sex) in the previous 6 months (*n* = 22) (Table 2).

A majority of all respondents, 65.6% (461/703) reported never having donated blood while 34.4% said they had previously donated blood. Of the 242 who said they had donated blood, 84.3% indicated that their donation occurred in or after 1984, the year that the MSM sexual deferral was introduced (*n* = 204). When asked if they had sex with another man in the 12 months before donating blood, 76% of those who donated in or after 1984 said no, while 23% said yes, and 1% did not answer.

### 3.1 Donor questionnaire

To be considered eligible to donate blood, 74.4% of participants (*n* = 523) were willing to be asked specific sexual behavior questions in a confidential interview, such as the last time they had sex and the precise type of sex they had. However, just over a quarter of participants, 25.3% said they would not be willing to be asked (*n* = 178) and 0.3% did not know or provided no response (*n* = 2). When we examine these responses by participants' reported openness about having sex with men, a majority of those who reported being extremely open (77%), somewhat open (73.4%), or not very open (77%) were willing to be asked specific sexual behavior questions. However, only 44.4% of those who reported not being open (“completely to myself”) said they were willing to be asked specific sexual behavior questions in a confidential interview and 55.6% said they were not (*p* < .05).

When asked if they would honestly answer sexual behavior questions if their eligibility to donate blood considered additional sexual behavioral factors instead of how recently they had sex with another man, a majority of participants said yes (71.3%). While 3% said that they would not answer honestly, just over a quarter (25.7%) did not know or provided no response. When we examine these responses by participants' reported openness about having sex with men, a majority of those who were extremely open (74.6%), somewhat open (68.8%), or not very open (74.3%) said they would answer honestly. However, only 44.4% of those who reported not being open said they would answer honestly (*p* < .01).

### 3.2 Understanding testing and transmission

Most participants (84.1%) reported that they had heard about the WP for detecting new HIV infections. When asked how long they believed the WP was for the methods currently used to screen donated blood, 30.7% said either “less than 1 week” or “between 1 week and 1 month” (61%) and just 8.3% said “between 1 and 3 months.”

When asked whether donated blood from a person with HIV who had an undetectable viral load could transmit HIV by transfusion, just under half (48.4%) correctly said yes. However, when we examine these responses by participants' reported openness about having sex with men, a majority (74.1%) of those who were not open (“completely to myself”) believed that the blood could transmit HIV compared to 45.9% of those “not very” open, 52.9% of those “somewhat” open, and 43.4% of those “extremely” open (*p* = .01).

## 4 DISCUSSION

Our results indicate that to donate blood, Australian gbMSM are generally accepting of more detailed questions regarding their recent sexual behaviors, although less so if they are not open about their sexuality. The Nucleic Acid Test (NAT) WP in blood donation testing is dependent on testing method (pooled or individual donation) and influenced by other factors such as PrEP use. Australia uses a combination of individual donation (ID‐NAT) and 16 donation pooled NAT (MP‐16) with estimated WPs of 5.4 and 8.5 days, respectively. Considering this, participant responses in respect of the WP duration suggest that most have a good understanding of the approximate WP duration. The transmissibility of donated blood from HIV+ individuals with an undetectable viral load was lesswell understood, with about half incorrectly stating that such blood would not be capable of transmitting HIV.

To donate blood, the majority (74%) of our respondents were comfortable with being asked more intrusive questions about the timing and nature of their most recent sexual contact, which has important implications for possible future policies centered on more detailed sexual activity questions. By comparison, respondents to the 2014 US NBS survey of MSM were slightly less comfortable with 59%–63% somewhat or strongly agreeing that they would be willing to be asked more detailed questions.^41^

In Canada, a large survey of the general blood donor population also identified discomfort to some potential new screening questions. The percentages of donors who were uncomfortable with individual candidate questions ranged from 6.5% to 17.2%, with the highest figure being for questions about having anal sex.^42^ Qualitative interviews with donors who indicated discomfort, revealed that they felt the questions were too personal and that they were unclear of the safety benefit. The authors concluded that implementing alternative questions would result in substantive deferrals, and that other policies such as using an MSM capture question to ask additional questions only to MSM donors should be considered.^42^ A subsequent large survey of Canadian blood donors assessing responses to novel sexual activity‐based screening questions implemented in the UK in 2021 found that while 20% reported feeling uncomfortable answering questions about higher‐risk sexual behaviors, only 2% said it would stop them from donating. The authors concluded that potential donor loss from implementing the UK questions could be compensated by newly eligible gbMSM and encouraging donation from infrequent donors.^43^

Two‐thirds of men surveyed had sex in the last 6 months with a non‐relationship partner and almost all (93.2%) had anal sex. If our survey is representative of the Australian gbMSM population, our results suggest any candidate approach that directly excluded for anal sex alone with more than one partner would prevent the majority of Australian gbMSM from donating. A gender‐neutral approach asking all donors about anal sex will also exclude a proportion of heterosexual donors. In a survey of UK participants 5% overall (3% men and 2% women) had anal sex in the last 3 months and 6% in the last 6 months.^44^ Similarly, in a Canadian blood donor study, slightly less, 3.7% of donors disclosed anal sex in the last 3 months.^42^

Of the men in our study who reported being HIV negative and on PrEP, 7.2% reported having no nonmonogamous sexual partners in the previous 6 months. Lifeblood's current deferral for PrEP use in the past 3–12 months would preclude donation from those men even if they had abstained from sex in the last 3 months (i.e., met Lifeblood's 3‐month MSM contact deferral). Lifeblood's PrEP deferral was initially “precautionary” and based on the then “theoretical” blood safety risk associated with the extension of the HIV RNA and serology window periods.^29^ PrEP deferrals now apply in a number of countries, including some that have transitioned to gender neutral screening approaches (e.g., UK, Netherlands, and France). A recent case report of a non‐compliant, Brazilian HIV positive donor retrospectively identified as having taken PrEP for 30 days pre‐donation confirms this blood safety risk.^45^

A sizeable majority (85%) of gbMSM in this survey had heard of the WP and their perception of its duration for donation screening tests was resonably accurate. Lifeblood's current HIV NAT has a WP of 5.4 (5–8) days for ID NAT and 8.5 days for MP‐16, therefore participants selecting “less than 7 days” (31%) as well as those responding, “between 7 days and 1 month” (61%) can be considered correct. With the caveat that 7 days to 1 month is a wide range, this suggests that in general Australian gbMSM have a sound understanding of the HIV WP in the context of donation testing. While the ability to accurately risk‐assess is important given evidence that some donors may not answer targeted sexual activity risk questions based on the question content, but rather their overarching perception of “is my blood safe today?,” other factors in addition to the HIV WP (e.g., sexual timing recall accuracy) are considered when determining eligibility in the context of transfusion safety.^46^

Given the global expansion of the Undetectable equals Untransmittable (U=U) HIV public health prevention campaign, we sought to determine if gbMSM understood that this is specific for sexual transmission. Lifeblood authors have previously highlighted that because of the much larger inoculum and parenteral route of transmission, U=U is not valid for transfusion, constituting a potential risk that HIV+ individuals might donate assuming their blood is safe.^37^ About half of the gbMSM surveyed thought that blood from an HIV infected person with an undetectable viral load had the potential to transmit should their blood be transfused. Those “not open” were significantly more likely than those completely open to correctly consider that transfusion was a risk even when the person's viral load was undetectable. This might reflect less‐than‐optimal understanding of the question, or that men who are not “out” have comparably less knowledge of the U=U campaign.

While “gender‐neutral” approaches to risk assessment‐based questioning targeting recent anal sex and new sexual partners potentially expand eligibility to a new group of gbMSM, there remains a not‐insubstantial proportion who would continue to be ineligible should this change occur. In particular, the almost 32,000 people in Australia, the vast majority being gbMSM accessing PrEP in the prior 12 months at the end of 2020^47^ would remain ineligible for whole blood donation until 12 months after their last dose. In addition, a significant proportion of MSM expressed discomfort with answering specific sexual behavior questions, with less than half of those who reported not being open saying that they would answer honestly. Therefore, Lifeblood contends that a plasmapheresis program for MSM restricted to manufacturing plasma‐derived blood products and not requiring any questions relating to sexual practices offers advantages over a gender‐neutral approach from the perspective of inclusivity, blood safety and donor experience, and particularly since more plasmapheresis donations are now collected in Australia than whole blood to meet expanding demand—predominantly for immunoglobulin. The inherent pathogen inactivation capacity of the fractionation process^48^ would even allow consideration of plasmapheresis donation by MSM on PrEP, without any deferral period. Another advantage of a plasma program is that it would not defer currently eligible heterosexual donors for anal sex, which by itself is not a known significant risk factor for HIV and has been demonstrated to be safe in that currently accepting these donors has not resulted in a HIV transfusion‐transmission in Australia. This would be problematic because of the sufficiency loss, and also because of the ethical validity of deferring heterosexual donors who are currently eligible because their HIV risk is acceptably low.

Limitations include the data being self‐reported and vulnerable to social‐desirability bias, with the proportion of gbMSM who were interested in donating blood possibly higher or lower than reported here. Moreover, a self‐reported history of blood donation could not be verified in this sample. Our study did not take into consideration the time of last donation relative to the time of last sexual contact with another man. Additionally, participants were predominantly recruited from online social networking sites; this may limit the generalizability of the study results. Regarding the WP duration question, it is possible that those taking PrEP may have factored in some delay to account for PrEP.

Our study indicates that Australian gbMSM are generally aware of the approximate HIV WP duration in the context of blood donation testing, important for their ability to correctly self‐assess their HIV risk. However, almost half of participants incorrectly assessed the transmissibility by blood from an HIV positive person with an undetectable viral load, which suggests the need for a targeted education campaign. While the majority seem comfortable with answering more detailed questions indicating they would do so honestly, men who are not open about their sexuality were less inclined to. Importantly, our results may not be generalizable, particularly in jurisdictions where gbMSM are less accepted due to social and cultural barriers and may be less open to declaring their sexual orientation. Therefore, we encourage other comparable studies to support local policy change. Any candidate gender‐neutral assessment strategy needs to consider the impact of asking questions about anal sex and new sexual partners, considering the consequences both for MSM and non‐MSM prospective donors. Given the ongoing need to expand plasma collections in Australia, creating a plasma for fractionation donation pathway for gbMSM offers advantages over a gender‐neutral approach. It would potentially constitute the most inclusive MSM policy worldwide, addressing a key sufficiency need with the inherent pathogen inactivation providing an additional safety plank.

## FUNDING INFORMATION

This study was funded by the Australian Research Council (ARC) grant number: RG132750. Support for NHMRC Fellowship holders was funded by the NHMRC Partnership Grant RG192651: *Protecting the blood supply against infectious disease by strengthening the evidence base that guides donor selection and screening policy 2018–22*. The Kirby Institute and the Australian Research Centre in Sex Health and Society receive funding from the Australian Government Department of Health. The National Drug and Alcohol Research Centre, UNSW is supported by funding from the Australian Government under the Substance Misuse Prevention and Service Improvements Grants Fund.

## CONFLICT OF INTEREST STATEMENT

The authors have disclosed no conflicts of interest.
